# Personality, Behavior and Environmental Features Associated with *OXTR* Genetic Variants in British Mothers

**DOI:** 10.1371/journal.pone.0090465

**Published:** 2014-03-12

**Authors:** Jessica J. Connelly, Jean Golding, Steven P. Gregory, Susan M. Ring, John M. Davis, George Davey Smith, James C. Harris, C. Sue Carter, Marcus Pembrey

**Affiliations:** 1 Department of Medicine, Division of Cardiovascular Medicine and Robert M. Berne Cardiovascular Research Center, University of Virginia, Charlottesville, Virginia, United States of America; 2 Centre for Child and Adolescent Health, School of Social and Community Medicine, University of Bristol, Bristol, United Kingdom; 3 Avon Longitudinal Study of Parents and Children, School of Social and Community Medicine, University of Bristol, Bristol, United Kingdom; 4 Department of Psychiatry, University of Illinois at Chicago, Chicago, Illinois, United States of America; 5 Department of Psychiatry and Behavioral Sciences, Developmental Neuropsychiatry, Johns Hopkins University School of Medicine, Baltimore, Maryland, United States of America; 6 Department of Psychiatry, University of North Carolina, Chapel Hill, North Carolina, United States of America; 7 Department of Psychology, Northeastern University, Boston, Massachusetts, United States of America; 8 Institute of Child Health, University College, London, United Kingdom; Hunter College, City University of New York (CUNY), CUNY School of Public Health, United States of America

## Abstract

**Background:**

It is assumed that the oxytocin receptor gene (*OXTR*) is associated with factors that are related to features of reproduction as well as the currently emerging fields of mood and emotional response.

**Methods:**

We analysed data from over 8000 mothers who participated in the Avon Longitudinal Study of Parents and Children (ALSPAC). We determined reproductive, emotional and personality differences related to the two SNPs rs53576 and rs2254298 of the oxytocin receptor gene to determine whether there was evidence in this population for: (i) associations with emotional and personality differences, and (ii) behavioural or environmental links with these SNPs using a hypothesis free approach with over 1000 types of exposure.

**Results:**

Our analyses of 7723 women showed that there were no differences in 11 mood, social or relationship characteristics associated with the rs2254298, and just one with rs53576 (with emotional loneliness) – one statistically significant out of 22 tests is no more than would be expected by chance. There were no interactions with childhood abuse. Using a hypothesis-free approach we found few indicators of environmental or behavioural differences associated with rs2254298, but there was an excess of associations with eating habits with rs53576. The findings included an association with dieting to lose weight, and habits typical of bulimia for the women with GG. The nutrition of the women also showed negative associations of the GG genotype with 13 nutrients, including vitamins D, B_12_ and retinol, and intake of calcium, potassium and iodine.

**Conclusions:**

We conclude that this large database of pregnant women was unable to provide confirmation of the types of personality associated with these two *OXTR* SNPs, but we have shown some evidence of eating differences in those with GG on rs53576. Confirmation of our hypothesis free associations using other data sets is important.

## Introduction

There are increasing numbers of publications, particularly in the field of neuroscience, that claim that an individual's oxytocin levels and sensitivity to oxytocin are linked to his/her social and emotional responses – and that this is true both of animal responses, particularly in the prairie vole [1], and of humans [2]. In controlled trials oxytocin has been administered as a nasal spray, with beneficial positive effects on mood and social behaviour [3]. However there are also important variations in sensitivity to oxytocin that are assumed to result from genetic and epigenetic variation in the oxytocin receptor gene (*OXTR*).

The original hypothesis that variation in the *OXTR* gene was associated with deficits in social behaviour arose from studies of individuals, usually males, with autism spectrum disorders, which are defined in part by deficits in social behaviour. These genetic studies were first conducted in 195 family trios including an offspring with autism in a Han population in China [4]. The study found that both the SNPs rs53576 and rs2254298 were associated with autism. A subsequent much smaller study of 57 trios of European descent in the United States failed to replicate the findings [5]. In spite of the complexity of the outcomes and the modest size of the studies, interest has grown in the broader hypothesis that genetic variation in the *OXTR* gene could predict sensitivity to social cues [6] or to oxytocin-based therapeutics for autism and schizophrenia [7]. The literature concerning the SNP that is most studied (rs53576) has usually compared the GG individuals with AG and AA combined. Various studies have shown that individuals with the GG genotype demonstrate increased maternal sensitivity to her child [8], decreased attachment related anxiety – but only in women [9], increased empathy defined as the ability to read emotion in the eyes [10], decreased stress reactivity [10], increased optimism, mastery and self-esteem [11], lower symptoms of depression [11], and independently rated pro-social behaviour [12]. Indeed there are blogs on-line created by individuals who are GG on rs53576 claiming that they are extremely empathetic in consequence [13]. However some studies have failed to find associations with adult attachment [14], social preferences [15] or optimism [16] with this SNP.

There is less evidence for the SNP rs2254298, although Feldman and colleagues^2^ have shown that this genotype is associated with plasma levels of oxytocin (individuals with GG having lower levels). Brüne [17] suggests that variation at rs2254298 is associated with sociability and a differential risk of depression and anxiety depending on early environmental exposures. In a review of the literature, Ebstein and colleagues [6] stated that “some but not all association studies suggest that polymorphisms in the *OXTR* gene contribute to social behaviour in both normal subjects as well as in individuals characterised by dysfunctional social cognition.” They identify the SNPs rs53576, and rs2254298 as being important in human social behaviour, including aggression.

However there are a number of difficulties in interpretation of the associations with the different SNPs in the *OXTR* gene: (a) few of the results compare data between men and women even though Tost and colleagues [18] have shown an intriguing relationship between rs53576 and neuroimaging results for males, but not females; others have also reported gender specific effects with rs53576 [9], [19], [20]; (b) rarely have the same outcomes been measured; (c) it is not known how often failure to replicate has resulted in failure to publish, and (d) there is the issue of small numbers in both original studies and replication samples with all the inherent problems of lack of power as outlined recently as a problem, particularly in neuroscience [21].

It is clear that both genetic and environmental factors are involved in the development of many outcomes. The major studies that have been undertaken to identify genetic variants associated with specific outcomes have usually used the GWAS approach with a case/control design [22]. Studies of the effects of the environment have been more focussed, and very few have assessed possible associations between genes and features of the environment apart from those known to be involved in the mechanism of an environmental effect such as alcohol [23] and nicotine [24]. However, an understanding of how polymorphic genes, particularly those implicated in behaviour, might impact on development and health can also be informed by looking for candidate gene associations within a large set of environmental exposures. This approach, dubbed the exposome [25], [26] mirrors the GWAS approach in being hypothesis free. An illustrative example of how genetic associations with environmental exposures can arise is given by a brief exposome scan with a SNP in the gene *ABCC11*, which is associated with variation in ear-wax. We found an association with deodorant use, and consequently with exposure to volatile air pollutants in those using aerosol deodorants – an association that is valid since as well as affecting the ear-wax, the *ABCC11* gene is associated with under-arm sweat [27].

In view of the lack of clarity in the literature on the *OXTR* gene variant associations and the potential benefit of an exposome scan, we report results from an opportunistic set of analyses on a large dataset. We use over 1000 environmental and phenotypic measures to assess possible relationships with two genetic variants (rs53576 and rs2254298) in a population sample of mothers using the Avon Longitudinal Study of Parents and Children (ALSPAC), and focus on two research questions: (a) do these women differ in their personality and emotional characteristics as described in the literature and (b) are there environmental differences in the women in the study associated with their polymorphisms on the SNPs rs53576 and/or rs2254298?

## Methods and Materials

### Study sample

The data used in these analyses were collected as part of the Avon Longitudinal Study of Parents and Children (ALSPAC), which was designed to assess the ways in which the environment interacts with the genotype to influence health and development [28]–[30]. Pregnant women, resident in the study area in southwest England with an expected date of delivery between 1^st^ April 1991 and 31^st^ December 1992, were invited to take part. About 80% of the eligible population did so [29], [30].

The women and their partners were sent a number of questionnaires during pregnancy. These elicited information on their family, their early childhood, adolescence and adulthood. The data analysed for this study comprises information collected up until the time of birth of the study child. As well as details collected from the questionnaires, we include details from the obstetric records (where available) and results of assays of the mothers' biological samples. Details of the questions asked, the ways in which they are coded and the frequencies of response can be found on the study web site: http://www.bristol.ac.uk/alspac/researchers/.

### The genetic variants

The oxytocin receptor gene (*OXTR*) is located on chromosome 3p25. It spans 19,206 basepairs (GRCh37/hg19 assembly) and contains 4 exons and 3 introns. Within this gene there are two variants that have been used more frequently than others: rs53576 and rs2254298 which are both situated in the third intron of the gene. They have not been found to have any clear functional impact on the gene although they may possibly be in linkage disequilibrium with a yet unidentified exonic SNP.

The rs53576 polymorphism was genotyped using the Illumina 660W-quad chip on the DNA of the mother. The genotype of variant rs2254298 was imputed using results from the GWAS analysis. We assessed the validity of the imputation using 459 DNA samples of these mothers, genotyped blind to the imputation results by one of us (JJC). The results were identical in 454 (98.9%) of women.

### Characteristics of the women

We assessed 11 characteristics of the study mother said to be associated with one or other of these two SNPs in the literature. For (a) positive affect we use the mother's rating of happiness in childhood; (b) negative affect used the mother's depressive symptoms at two stages of pregnancy; (c) sensitivity using the Boyce and Parker total interpersonal sensitivity inventory [31]; (d) emotional loneliness using the perceived social support scale [32]; (e) empathy using the empathy sub-section of the Boyce and Parker scale [31]; (f) attachment using the separation anxiety subsection of the Boyce and Parker scale [31]; (g) mastery using a locus-of-control scale based on that of Nowicki and Duke [33]; (h) self-esteem using the Bachman scale [34]; (i) stress reactivity using the Interpersonal awareness subscale of the Boyce and Parker measure [31]; (j) marital conflict using a scale relating to the frequency with which the mother argues and/or physically fights with her partner. The various measures used are described in more detail in Annex S1. All measures were administered during pregnancy with the exception of the self-esteem scale, which was administered 33 months after the birth of the child.

### Statistical approach

We treat the data in two different ways. First, we test the assumption that these two polymorphisms are associated with social personality characteristics of the woman. This uses traditional techniques to test the associations promulgated in the literature for associations in the predicted direction [35]. Since there have been some reports indicating that associations were stronger if there had been an adverse experience in the past [36], [37], we also tested each personality relationship for an interaction with reported abuse in childhood (any physical, emotional or sexual abuse reported by the woman).

Second, we analyse the environmental measures using an exposome analysis. We use all environmental and behavioural exposures identified in ALSPAC up until the time of delivery of the study baby. This includes aspects of the early childhood of the mother and of her parents. The approach is similar to that taken in genome wide association studies (GWAS), being hypothesis free. Rather than testing already formulated hypotheses, GWAS examines associations with as many as 600 thousand genetic markers for pre-determined statistical significance levels, and then attempts to replicate the findings in other studies [22]. In general, no adjustment is made for confounders. Here we assess about 1300 environmental and behavioural associations with the two genetic variants; for each variant the literature mainly reports a difference between the GG and the (A/G OR AA) types, and we therefore compare the two in regard to the various environmental outcomes. In this study, which is a hypothesis generator in regard to the environmental factors, we first assess the relationships categorising the P values<0.10, <0.05 and <0.01; we then assess whether the numbers of variables identified in this way occur more often than expected by chance. In particular we look for unexpected patterns of association. In order to ensure that we do not suffer from type II errors, we do not correct for multiple testing at this stage. In order to look for patterns, the environmental variables are divided and considered in fairly logical groupings.

## Results

Genotype results were available for rs53576 and rs2254298 for 8330 and 8340 women respectively; 99.9% of the population were white. For rs53576 the frequencies were 46.0%, 43.9% and 10.1% for GG, AG and AA respectively. For rs2254298 they were 79.3%, 19.3% and 1.4%. There was a positive correlation between the two genotypes (r = 0.118; P = 0.005) indicating that they were in linkage disequilibrium.

### Hypothesis related analyses: personality and mood characteristics of the women

In Table 1 are listed the relationships between the various measures of personality type. There were 22 tests and for only one was P<0.05 – i.e. no more than would have been expected by chance. This association with rs53576 indicated that the women with GG reported lower levels of emotional loneliness than those with the A allele. In regard to possible interaction with a history of abuse, this was tested comparing the 1241 women who had had a history of being abused in childhood with the rest of the population; of the 22 characteristics tested no interactions were significant at the 5% level (Table S1).

**Table 1:** Relationships^a^ between GG genotypes on two SNPs in the *OXTR* gene and personality characteristics of the mother (n = 7723).

	rs53576		rs2254298	
Personality characteristic	GG v [AG+AA]	P	GG v [AG+AA]	P
Stress reactivity	−0.13 [−0.34, +0.08]	0.232	+0.11 [−0.15, +0.37]	0.410
Need for approval	−0.09 [−0.25, +0.07]	0.276	−0.06 [−0.26, +0.13]	0.528
Attachment	+0.00 [−0.22, +0.21]	0.967	+0.00 [−0.27, +0.26]	0.976
Empathy	−0.05 [−0.25, +0.16]	0.664	−0.01 [−0.26, +0.24]	0.932
Fragile inner self	+0.04 [−0.09, +0.18]	0.537	−0.02 [−0.18, +0.14]	0.810
Sensitivity	−0.23 [−0.95, +0.50]	0.538	+0.01 [−0.88, +0.90]	0.982
Locus of control	+0.04 [−0.05, +0.14]	0.380	+0.02 [−0.09, +0.14]	0.682
Emotional loneliness	**−0.25 [−0.48, −0.02]**	**0.034**	**+**0.05 [−0.23, +0.33]	0.741
Self-esteem	−0.01 [−0.33, +0.30]	0.930	+0.35 [−0.04, +0.74]	0.080
Inter-partner aggression	−0.05 [−0.13, +0.03]	0.215	+0.01 [−0.09, +0.11]	0.833
Inter-partner affection	+0.14 [−0.06, +0.33]	0.164	+0.14 [−0.10, +0.37]	0.258

a The results shown comprise the (unstandardized) difference between the mean scores of the personality characteristic with the 95% confidence interval.

There were, in addition, 6 measures of negative affect measured relating to depressive, anxiety, somatic and malaise symptoms at each of two trimesters of pregnancy. Of the 16 comparisons with the SNPs, only one had a P value<0.10, which was considered no more than expected by chance.

### Hypothesis-free analyses: Relationships with the environment and behaviour

#### rs53576

The relationships between genotype GG and (AG+AA) of rs53576 and various groups of environmental and behavioural factors are delineated in Tables S2, S3, S4, S5, S6, S7, S8, S9, S10, S11. The results are summarised in Table 2. Of the 1284 variables considered 128.4 would be expected to be associated at the 0.10 level, 64.2 at the 0.05 and 12.8 at the 0.01 level. In fact, as can be seen, there were 157, 78 and 14 respectively observed. On the assumption that all the associations are statistically independent, one could conclude that there were more associations at the 0.10 level than would be expected by chance. We therefore explored the relationships with the particular groupings to determine the likelihood of specific types of environment being responsible for this finding.

**Table 2:** Results concerning the exposome.

		rs53576		rs2254298	
Table Number	Number of Variables	<0.10	<0.05 [<0.01]	<0.10	<0.05 [<0.01]
S2	49	6	0 [0]	5	3 [1]
S3	183	22	10 [0]	13	9 [1]
S4	239	22	14 [3]	23	15 [5]
S5	134	12	6 [2]	16	10 [1]
S6	162	16	6 [2]	16	10 [0]
S7	143	29^c^	22^c^ [4]	9	6 [0]
S8	74	17^b^	8^a^ [0]	9	6 [3]^a^
S9	78	9	3 [1]	5	3 [0]
S10	132	12	5 [1]	15	7 [3]
S11	90	12	4 [1]	12	5 [0]
Total	1284	157^a^	78 [14]	123	74 [14]

Significant groups are indicated with a lower case letter, number of observed compared with expected:

a P<0.05;

b P<0.001;

c P<0.0001.

Table number refers to tables in Supporting Information.

The grouping of the variables in the tables are as follows: S2 = the maternal grandparents; S3 = the mother in childhood; S4 = the mother's physical environment; S5 = the mother's biological and medical history prior to pregnancy; S6 = the history of the study pregnancy; S7 = maternal diet and feeding habits; S8 = maternal exposure to noise and social drugs; S9 = maternal social exposure, moods and personality; S10 = antenatal, labour and delivery details mainly abstracted from obstetric records; S11 = the newborn.

The major differences between the GG and the AG/AA group of mothers concern the results in regard to Tables S7 (diet and nutrition) and S8 (social drugs). The latter is concerned mainly with various aspects of smoking, the women with GG genotype were significantly less likely to have smoked pre-pregnancy [OR 0.91, 95% CI 0.82, 1.00], in the first trimester [0.87, 95% CI 0.78, 0.97], in the second trimester [0.87, 95% CI 0.77, 0.98], in the last 2 months of pregnancy [0.86, 95% CI 0.76, 0.97] and during labour [0.82, 95% 0.67, 1.01]. These 5 variables are strongly inter-related; for example, taking account of smoking in mid-pregnancy resulted in the other smoking variables ceasing to have a P value<0.10. The inter-relationships of these, and other smoking related variables can all be subsumed under one variable, and consequently the variables in S8 are contributing little to the overall pattern.

However in regard to the woman's eating habits there were major and significant differences over and above those that would be expected by chance (of the 143 items assessed, 29 were related at the 0.10 level compared with 14 expected by chance). These include an increase in the women with GG genotype reporting ever having made herself vomit [OR 1.28, 95% CI 1.04, 1.56] or used laxatives [OR 1.31, 95%CI 1.05, 1.63] to lose weight. These women were also more likely to report going on a diet to lose weight [OR1.10, 95% CI 1.00, 1.21]. In regard to the actual foods and drinks consumed they were significantly more likely to use skimmed or semi-skimmed milk, and eat salads; as a group they were less likely to eat meat pies and pasties, eggs, baked beans, cakes, puddings, chapattis or to use hard/soft margarine for frying. Calculation of the nutrition of these women indicates that they were particularly short of specific nutrients compared with women with an A allele (Table 3). In order to ensure that the women with signs of bulimia did not account for these findings, the analyses of the nutrients were repeated excluding those with a history of making themselves vomit or taking laxatives. The result was that 15 nutrients were now included with a P value<0.10 (Table S12) rather than the original 13; the variables now included were carbohydrates, riboflavin and phosphorus, and vitamin D was no longer qualified as P<0.10. In all instances the women with GG on rs53576 were consuming less.

**Table 3:** Comparison of daily nutrient intake of rs53576_GG women compared with those with an A allele – those with P<0.10 listed.

Nutrient	Effect size [95% CI]	P value
Non-milk extrinsic sugar (g)	−1.99 [−3.55, −0.43]	0.012
Sugar (g)	−2.29 [−4.05, −0.53]	0.011
Energy (MJ)	−94.2 [−186.1, −2.29]	0.045
Fat (g)	−1.26 [−2.34, −0.19]	0.021
Monounsaturated fat (g)	−0.43 [−0.80, −0.07]	0.021
Saturated fat (g)	−0.64 [−1.17, −0.11]	0.018
Cholesterol (mg)	−5.51 [−9.55, −1.48]	0.007
Vitamin D (µg)	−0.08 [−0.18, +0.01]	0.089
Vitamin B_12_ (µg)	−0.16 [−0.28, −0.04]	0.011
Retinol (µg)	−18.0 [−33.8, −2.30]	0.025
Calcium (mg)	−11.8 [−24.8, +1.33]	0.078
Potassium (mg)	−29.6 [−63.0, +3.84]	0.083
Iodine (µg)	−3.07 [−5.28, −0.86]	0.007

#### rs2254298

In contrast with rs53576, the SNP rs2254298 appears to be unrelated to the environmental and phenotypic features measured (Tables S2, S3, S4, S5, S6, S7, S8, S9, S10, S11 and Table 2). There was just one group of variables that were associated with the GG genotype more than would be expected by chance: that relating to Table S8 where 3 variables were associated at P<0.01 compared with 0.74 expected. The relationships concerned were: the frequency the woman was in a room with the radio on (P = 0.007); frequency with which mother was in a room where others were smoking (P = 0.004); and the frequency with which she worked in a smoky atmosphere (p = 0.007). For the first two of these there was not a clear trend with dose, and it is difficult to interpret the results as being meaningful. For the frequency with which the woman worked in a smoky atmosphere there was a trend such that those with the A allele were more exposed.

### Evidence for relationships with features of reproduction

As part of this analysis, we have included various descriptors of the women involved that may support or refute the findings in the literature. We assessed whether their past obstetric history, or the history of the current pregnancy, differed with genotype. There was only one association at P<0.010 with the past obstetric history compared with three expected from the two SNPs. For the history of the study pregnancy, of some note is the fact that GG in rs53576 was associated with a prolonged second stage of labour [P = 0.091] and a reduction in the likelihood of induction with syntocinon [P = 0.082]. For rs2254298 there was no association with a prolonged second stage [P = 0.479] or induction with syntocinon [P = 0.249].

## Discussion

This study has been undertaken with two aims: (i) to assess whether the two genetic variants quoted frequently in the literature were in any way associated with mood, personality or other social characteristics in this population comprised only of pregnant women; (ii) to determine whether the genotypes were associated with other factors that resulted in a change in the maternal environment.

### Aim (i)

This study was devised in order to confirm (or otherwise) whether the reported associations with social attitudes and characteristics of the woman were related in any way to two of the genetic markers of *OXTR* that were most often quoted in the literature as having such effects (rs53576 and rs2254298). However we found no indication at all that there were links with these markers.

This raises major questions as to why we have found no such associations in spite of having a large representative population of women. The possibilities are: (a) pregnant women are not an appropriate study group as the hormones to which they are exposed at this time may mask underlying features of personality; (b) women in general may not be appropriate – one study has shown a male-specific relationship between the SNP rs53576 and differences in brain morphology [18], and one small study found associations with positive affect that was restricted to males [20]; it may also be relevant that the only large population study to test one of the findings (optimism) with this genetic variant, the American Nurses Health Study, was unable to confirm the original finding – all participants were female [16]. Though females have a higher risk of developing disorders related to stress, males are at higher risk for development of early life psychiatric disorders such as autism and ADHD. The differential impact of the oxytocin system may contribute to this distinction. (c) The studies that have been published tend to be small, and rarely use outcome variables that are comparable [21]. (d) Other possible reasons for our failure to replicate concern the possibility that associations between the genetic markers and social/emotional outcomes are contingent on the environment. However, we have compared results among women who had had a history of abuse in childhood with those with no such history, but found no difference in relationships (Table S1). (e) The contribution of the epigenome has not been considered. (f) However the most likely explanation of our failure to replicate is publication bias – whereby results that are not statistically significant or in an unexpected direction are never published.

### Aim (ii)

Our second aim was to assess whether there were any unexpected associations using a hypothesis free approach. This exposome approach as originally conceived [25], [38] mainly assumes that biomarkers would be the measures of the environment, but Peters and colleagues have recently pointed out a number of problems with this, including the fact that relevant environmental exposures such as noise, heat or electromagnetic fields do not have such direct correlates as metabolites or protein adducts, although there is important evidence linking them with health effects [39]. We suggest that there is no reason why the traditional measures of the environment as used in classical epidemiology should not be used. Indeed Wild has recently expanded the concept to state that environment should be considered in the broadest context of non-genetic; he also suggests that the exposome should cover life-long exposure history [26]. This implies consideration of environmental exposures from conception onwards, as performed in our study of ALSPAC mothers by including features of their own parents' lives (study grandparents of Table S2). Ideally the exposome data should start even before conception of the index individual, since the father's germ cells are developed during his childhood prior to the onset of puberty and the mother's eggs developed during her fetal life, and consequently the environment at each phase of the individuals' parents' life may result in changes to the germ cells – with consequent changes to the index individual.

Distinguishing between exposome and phenome scans can be difficult, as shown by the following examples: (i) we have shown that a SNP in the *ABCC11* gene is associated with the use of deodorant; this could be considered a phenotype (as an indicator of tendency to sweat under-arms), or as an indicator of exposure to the chemicals in deodorants [27]. (ii) Nicotine metabolism is strongly related to genetic variants such as CYP2A6; individuals with particular variants in this gene are protected from dependency on nicotine and consequently smoke fewer cigarettes, thus resulting in a different environment [40]. Thus, for many of the variables we consider as environmental in this study, we could have described them as phenotypical.

The major finding from our hypothesis free exposome/phenome approach [41] has been the demonstration of a substantial change in the dietary choices made by the study mother. This appears to result in a reduction in nutrients that may impact on her own health and well-being as well as that of the developing fetus. Although overall the fact that the GG women were consuming fewer calories, particularly in regard to less sugar, fats and cholesterol may be of little consequence (and even of advantage), it is of concern that they had lower intakes of vitamins D, B_12_ and retinol, and of nutrients calcium, potassium and iodine. Such deficiencies may put the fetus at some risk. This is particularly relevant to iodine intake in Britain since the population is generally deficient, and it has been shown that low maternal iodine in the first trimester has a long term effect on the cognitive outcome of the fetus [42]. It is important that the results be replicated – but meanwhile the study of Lawson and colleagues [43] who studied groups of women with anorexia and controls and reported that they found higher oxytocin secretion in response to a meal associated with higher levels of disordered eating psychopathology in women with active and weight-recovered anorexia nervosa may be of relevance. They suggested that oxytocin has appetite-regulating functions.

### Strengths and limitations

There are a number of strengths to this study – first it is considerably larger than any other that has looked at the relationship between the *OXTR* SNPs and emotional and mood outcomes. Second, the data were collected prospectively from a large geographically defined population, and consequently not influenced by characteristics that might bias the responses. Third, although much of the data have been collected by self-report using structured questionnaires which may be criticised, where comparisons have been able to be made with biological sample assays, high correlations have been found.

The limitations, as already outlined are that the study comprises pregnant women only, and cannot be extrapolated to non-pregnant women or to men. In addition, the associations found with the eating habits of the mother must be considered as hypothesis generating. It is important that they be tested in another dataset.

### Conclusion

We were unable to confirm any of the reports in the literature of associations between two *OXTR* genetic variants and characteristics, moods or relationships of the women in this study. The possibility remains that there still are associations between the *OXTR* SNPs contingent on the environment, or among males rather than females. Meanwhile it may be useful to be aware that there are consistent relationships between rs53576 and eating behaviours in this study.

## Supporting Information

Annex S1 (DOCX)

Table S1 (DOCX)

Table S2 (DOCX)

Table S3 (DOCX)

Table S4 (DOCX)

Table S5 (DOCX)

Table S6 (DOCX)

Table S7 (DOCX)

Table S8 (DOCX)

Table S9 (DOCX)

Table S10 (DOCX)

Table S11 (DOCX)

Table S12 (DOCX)
