# Supplementary material for: Personality, Behavior and Environmental Features Associated with OXTR Genetic Variants in British Mothers
Source: PLoS One. 2014 Mar 12;9(3):e90465. doi: 10.1371/journal.pone.0090465 (PMC3951216; doi:10.1371/journal.pone.0090465)
Supplement: Annex S1 — (DOCX) [file pone.0090465.s001.docx]

**Annex S1. The personality and mood measures used**

***The Boyce and Parker personality measures***

The 36 items in this scale formed the Inter-Personal Sensitivity Measure (IPMS) devised by Boyce & Parker[1]. This measure has 5 sub-scales: inter-personal awareness, need for approval, separation anxiety, timidity and fragile inner-self. Each item has 4 categories of response in which the respondent indicates whether the description provided in each item is ‘very like’, ‘moderately like’, ’moderately unlike’ or ‘very unlike’ themselves. Initial studies of the measure have indicated that it has a satisfactory split off and test-retest reliability and has concurrent validity with psychiatric interview. The measure correlates high with neuroticism scales from the Eysenck Personality Questionnaire (EPQ)[2] but has been found to be a better predictor of post-natal depression scores as measured by the EPDS[3]. The scores comprise the responses to the following:

*Interpersonal awareness*

The 7 statements were: *I worry about the effect I have on other people; I feel uneasy meeting new people; I worry about being criticised for things I have said or done; If someone is critical of something I do, I feel bad; If someone upsets me, I am not able to put it easily out of my mind; I worry about what others think of me; I care about what people feel about me.* High scores indicated very concerned about others feelings about herself.

*Need for approval*

This scale was calculated from the responses to 8 statements: *I feel secure when I'm in a close relationship; After a row with a friend, I feel uncomfortable until I have made peace; I always notice if someone doesn't respond to me; I feel that people generally like me; I will go out of my way to please someone I am close to; I feel happy when someone compliments me; I can make other people feel happy; I feel hurt when someone is angry with me.* High scores indicate very much in need of reassurance

*Separation anxiety score*

The 8 statements comprising this scale were: *I feel insecure when I say goodbye to people; I feel anxious when I say goodbye to people; I worry about losing someone close to me; I can only believe that something I have done is good when someone tells me; I fear that my feelings will overwhelm me; I always expect criticism; I can never be really sure if someone is pleased with me; I feel others do not understand me.* High scores indicate higher levels of insecurity.

*Empathy score (also known as the Timidity score)*

This score, which should be considered an empathy measure, was calculated from the responses to the 8 statements: *I avoid saying what I think for fear of being rejected; I don't get angry with people for fear that I may hurt them; I am always aware of how other people feel; I will do something I don't want to do rather than offend or upset someone; I find it hard to get angry with people; I worry about criticising people; I am never rude to anyone; I worry about hurting the feelings of other people.* Higher scores indicate more empathetic.

*Fragile inner-self score*

There were just 5 statement making up this scale: *If others knew the real me, they would not like me; If other people knew what I am really like, they would think less of me; I don't like people to really know me; I don't feel happy unless people I know admire me; My value as a person depends enormously on what others think of me.* Higher scores indicate that the individual is very uncertain of her own self-worth.

*Total inter-personal sensitivity score*

This comprised the sum of the 5 scales above.

***The Edinburgh Postnatal Depression Score (EPDS)***

The 10 items D24-D33 formed the depression scale of the Edinburgh Post-Natal Depression Scale (EPDS) developed by Cox *et al*[3]. The items in this scale were specifically chosen by the authors because they did not involve somatic items. Each question had 4 response categories scored from 0 to 3 and referred to the feelings of the mother in the past week. Although the measure was developed specifically for use with puerperal women, none of the 10 items is specific to the postnatal experience. The principle feature of the scale that designates it as a post-natal scale is that it does not include somatic items because of the possibility of confounding somatic symptoms of depression with normal physiological symptoms at this time. The feature of the scale was a major factor in its selection for our study, which aims to measure depression during pregnancy and the post-partum years. During pregnancy, there is the possibility too of confounding normal physiological symptoms with those of depression. Both our own pilot studies and the study of Murray and Carrothers found the measure to be acceptable to respondents, producing high completion rates with little evidence of response error[4]. Validation of the scale during pregnancy, the post-partum period and early parenthood has been examined using standardised psychiatric interviews as the validating measures and shown to have high sensitivity and specificity. The higher the score, the greater the level of depressive symptoms.

***The Crown-Crisp Experiential Index (CCEI)***

Although the total score of the 48 items of the Crown-Crisp Experiential

Index[5] in the original index has been shown to be a useful measure of psycho-neurotic pathology in the community, the need to limit the number of items and ALSPAC’s specific interest in depression and anxiety guided the selection of the 23 items relating to free-floating anxiety, depression and somaticism. Indeed most studies using the CCEI, including those of the original authors have in fact focused on the sub-scales and it has been used in this way in the study of mental health of mothers during pregnancy and the post-natal year. The three sub-scales had varying styles of response, some being a two-point yes/no scale, while others had 3-point categories. We modified the response categories for our study so that each item had four consistent response categories in which the respondent indicated frequency of symptoms from ‘never’ to ‘very often’. Scoring was such that the higher the score the greater the level of mental distress. The sum of the three sub-scales is named the Malaise score.

*Marital relationship*

A set of 9 questions relating to the marital relationship were developed for ALSPAC, based on the work of Quinton & Rutter[6] and Stanley[7]. Each was given 5 possible responses: almost always; often; sometimes; rarely; never. They were used to create two scales as shown:

*Affection score*

6 items were used as follows: *Is your partner affectionate toward you? Does your partner listen to you when you want to talk about your feelings? Does your partner talk to you about his problems and feelings? Do you enjoy the company of your partner? Does your partner show his approval of you? Do you behave affectionately toward your partner?* This was scored such that the lower the score the greater the affection.

*Aggression score*

Just 3 items were used for this scale: *Does your partner get angry with you? Do you have arguments with your partner? Do you get angry with your partner?* Scoring was such that the lower the score the greater the aggression.

***Parent bonding instrument***

The original Parental Bonding Instrument[8] had been adapted by Gamsa[9] to reword the statements that had produced double negatives in the original. During the course of piloting it became obvious that our mothers were unhappy with the original options for responses (very like, moderately like, moderately unlike, very unlike) and they have been changed as shown below. In addition 3 questions were omitted since they were almost identical to other questions in the scale and caused considerable annoyance to participants. Thus the instrument used comprised 22 items, each of which had possible responses: never; sometimes; usually. From these two scales were derived – a maternal care and an overprotection score. For reasons of space we did not enquire about the mother’s relationship with her father in this way.

*Maternal care score*

This score included items that measured the study mother’s perception of the relationship she had with her own mother – the higher the score the warmer the relationship.

*Maternal over-protective score*

This scale measured the degree to which the study mother felt that her own mother had been over-protective and failed to allow her to make her own choices in childhood. The higher the score the more oppressive was the relationship.

***Locus of control***

Locus of control of reinforcement has been defined as the perception of a connection between one’s actions and their consequences[10]. People who believe that an outcome is largely contingent upon their own behaviour are seen as having a more *internal* locus of control, whereas those who believe that luck, fate, chance or powerful others largely determine an outcome are considered to be more *external*. Measures of internality and externality have been shown to be associated with a number of different factors, including academic achievement, psychological well-being and beliefs[11,12].

The locus of control measure used in the present study is a shortened version of the AINSIE, the adult version of the Nowicki-Strickland Internal-External locus of control scales. The AINSIE comprises 40 items in a yes/no format, which assess perceived control[13]. This was chosen over other scales more specifically related to perceived control over health, as it was considered that this more generalised scale would relate to other factors in addition to health outcomes. The version used in the present study comprises 12 of the original 40 items, chosen after factor analysing the AINSIE administered as a pilot to 135 mothers, with the agreement of Professor Steven Nowicki. From the 12 questions a ‘locus of control score’ was derived, ranging from 0 to 12, with median 4. The higher the score the more external the locus of control was.

***Social network and social support***

The social network scale comprised 10 questions, which ascertain the extent of the mother’s social network. They were devised for the study based on a previous inventory used in Western Australia[7]. The questions were extensively piloted and found to obtain high completion rates. It was a factual scale enquiring about the number of friends, relatives, etc that the mother had contact with in the previous month.

The social support scale was used to measure emotional loneliness; it also comprises a 10-item set of questions. It is more subjective and measures perceived social support from family, friends and official agencies. It was adapted by the ELSPAC team from work carried out in Greece by Thalia Dragonas. The items present statements relating to emotional, financial and instrumental support to which the respondent is offered 4 response categories: ‘I never feel this way’, through to ‘This is exactly how I feel’. The alpha for this scale was somewhat low (0.58), but it should be noted that there was no presumed overlap among the types of support assessed or the persons providing the support[14].

***Self-esteem***

The mothers in ALSPAC only had their self-esteem measured when the child was aged 33 months. The Bachman Self-Esteem Scale was used[15]. This has been reported as having a good test-retest reliability (r=0.88), and content and construct validity[16]. The score is on a scale of 0 to 40. A high score corresponds to high self-esteem.

**References**

1. Boyce P, Parker G (1989) Development of a scale to measure interpersonal sensitivity. Aust N Z J Psychiatry 23: 341–351.

2. Eysenck HJ, Eysenck SBG (1975) Manual of the Eysenck Personality Questionnaire (junior and Adult). Sevenoaks, Kent: Hodder & Stoughton.

3. Cox JL, Holden JM, Sagovsky R (1987) Detection of postnatal depression. Development of the 10-item Edinburgh Postnatal Depression Scale. Br J Psychiatry 150: 782–786.

4. Murray L, Carothers AD (1990) The validation of the Edinburgh Post-natal Depression Scale on a community sample. Br J Psychiatry 157: 288–290.

5. Crown S, Crisp AH (1979) Manual of the Crown-Crisp Experimental Index. London: Hodder & Stroughton.

6. Quinton D, Rutter M (1988) Parenting Breakdown. Gower Publishing Company. 1 ESRC/DHSS. Aldershot:Avebury.

7. Honnor MJ, Zubrick SR, Stanley FJ (1994) The role of life events in different categories of preterm birth in a group of women with previous poor pregnancy outcome. Eur J Epidemiol 10: 181–188.

8. Parker G, Tupling H, Brown LB (1979) Parental Bonding Instrument (PBI). Br J Psychol S2:1-10.

9. Gamsa A (1987) A note on a modification of the Parental Bonding Instrument. Br J Med Psychol 60 ( Pt 3): 291–294.

10. Rotter JB (1966) Generalized expectancies for internal versus external control of reinforcement. Psychol Monogr 80: 1–28.

11. Lefcourt HM (1982) Locus of control: Current trends in theory and research. *2^nd^ Edition*. New Jersey: Lawrence Erlbaum.

12. Lefcourt HM (1984) Research with the locus of control construct. *Volume 2. Development and Social Problems.* New York: Academic Press.

13. Nowicki S Jr., Duke MP (1974) A Locus of Control Scale for Noncollege as Well as College Adults. Journal of Personality Assessment 38: 136–137.

14. O'Connor TG, Thorpe K, Dunn J, Golding J (1999) Parental divorce and adjustment in adulthood: findings from a community sample. The ALSPAC Study Team. Avon Longitudinal Study of Pregnancy and Childhood. J Child Psychol Psychiatry 40: 777–789.

15. Bachman JG, O'Malley PM (1977) Self-esteem in young men: a longitudinal analysis of the impact of educational and occupational attainment. J Pers Soc Psychol 35: 365–380.

16. Medora NP, Goldstein A, Hellen von der C (1993) Variables related to romanticism and self-esteem in pregnant teenagers. Adolescence 28: 159–170.
