# Supplementary material for: Personality, Behavior and Environmental Features Associated with OXTR Genetic Variants in British Mothers
Source: PLoS One. 2014 Mar 12;9(3):e90465. doi: 10.1371/journal.pone.0090465 (PMC3951216; doi:10.1371/journal.pone.0090465)
Supplement: Table S1 — (DOCX) [file pone.0090465.s002.docx]

# Table S1. Interactions (P values) between abuse in childhood and OXTR SNP genotype (GG v AG/AA)

| **Personality characteristic** | **rs53576** | **rs2254298** |
| --- | --- | --- |
| Stress reactivity | 0.399 | 0.942 |
| Need for approval | 0.476 | 0.778 |
| Attachment | 0.103 | 0.847 |
| Empathy | 0.823 | 0.124 |
| Fragile inner self | 0.329 | 0.451 |
| Sensitivity | 0.253 | 0.639 |
| Locus of control | 0.545 | 0.198 |
| Emotional loneliness | 0.376 | 0.076 |
| Self-esteem | 0.782 | 0.640 |
| Inter-partner aggression | 0.844 | 0.908 |
| Inter-partner affection | 0.707 | 0.804 |

Note: abuse was coded if a parent of the study mother had been emotionally or physically cruel and/or the study mother had been sexually abused in childhood.
