# Supplementary material for: Personality, Behavior and Environmental Features Associated with OXTR Genetic Variants in British Mothers
Source: PLoS One. 2014 Mar 12;9(3):e90465. doi: 10.1371/journal.pone.0090465 (PMC3951216; doi:10.1371/journal.pone.0090465)
Supplement: Table S2 — (DOCX) [file pone.0090465.s003.docx]

|  |  |  | **rs53576** | | **rs2254298** | |
| --- | --- | --- | --- | --- | --- | --- |
| **Table Number** | **Topic** | **Number of Variables** | **<0.10** | **<0.05 [<0.01]** | **<0.10** | **<0.05 [<0.01]** |
| MGM.1 | Maternal grandmother: basic [5504-7278] | 5 | 1 | 0 [0] | 0 | 0 [0] |
| MGM.2 | Maternal grandmother: health [7234-7295] | 15 | 2 | 0 [0] | 4 | 2 [1] |
| MGM.3 | Maternal grandmother: sociodemographic [4091-7304] | 6 | 1 | 0 [0] | 0 | 0 [0] |
| MGF.1 | Maternal grandfather: basic [5234-7149] | 5 | 1 | 0 [0] | 0 | 0 [0] |
| MGF.2 | Maternal grandfather: health [7148-7295] | 15 | 1 | 0 [0] | 1 | 1 [0] |
| MGF.3 | Maternal grandfather: sociodemographic [6023-6166] | 3 | 0 | 0 [0] | 0 | 0 [0] |
| **TOTAL** |  | **49** | **6** | **0 [0]** | **5** | **3 [1]** |

Table S2. The maternal grandparents

Note: the range of the number of valid observations by topic is shown in square brackets
