# Supplementary material for: Personality, Behavior and Environmental Features Associated with OXTR Genetic Variants in British Mothers
Source: PLoS One. 2014 Mar 12;9(3):e90465. doi: 10.1371/journal.pone.0090465 (PMC3951216; doi:10.1371/journal.pone.0090465)
Supplement: Table S3 — (DOCX) [file pone.0090465.s004.docx]

Table S3. The mother in childhood

|  |  |  | **rs53576** | | **rs2254298** | |
| --- | --- | --- | --- | --- | --- | --- |
| **Table Number** | **Topic** | **Number of Variables** | **<0.10** | **<0.05 [<0.01]** | **<0.10** | **<0.05 [<0.01]** |
| M.1 | The mother at birth [4587-8288] | 5 | 1 | 1 [0] | 0 | 0 [0] |
| MC.2 | The mother in infancy [6168-8330] | 10 | 3 | 1 [0] | 0 | 0 [0] |
| MC.3a | The mother <5y: accidents [7295] | 12 | 0 | 0 [0] | 2 | 2 [0] |
| MC.3b | The mother <5y: household [7295] | 11 | 1 | 0 [0] | 1 | 0 [0] |
| MC.3c | The mother <5y: traumatic events [7004-7425] | 6 | 0 | 0 [0] | 0 | 0 [0] |
| MC.4a | The mother 6-11y: accidents [7295] | 12 | 0 | 0 [0] | 1 | 1 [0] |
| MC.4b | The mother 6-11y: household [7295] | 12 | 2 | 1 [0] | 0 | 0 [0] |
| MC.4c | The mother 6-11y: traumatic events [7182-7510] | 6 | 1 | 0 [0] | 2 | 2 [0] |
| MC.4d | The mother 6-11y: other [7293-7484] | 4 | 0 | 0 [0] | 0 | 0 [0] |
| MC.5a | The mother 12-15y: accidents [7295] | 12 | 2 | 1 [0] | 1 | 0 [0] |
| MC.5b | The mother 12-15y: household [7295] | 12 | 1 | 1 [0] | 0 | 0 [0] |
| MC.5c | The mother 12-15y: traumatic events [7182-7540] | 6 | 2 | 1 [0] | 0 | 0 [0] |
| MC.5d | The mother 12-15y: other [7293-7484] | 2 | 0 | 0 [0] | 0 | 0 [0] |
| MC.6a | The mother <17y: traumatic events [7251] | 34 | 4 | 3 [0] | 2 | 1 [0] |
| MC.6b | The mother <17: school & environment [6956-7295] | 23 | 4 | 1 [0] | 1 | 1 [0] |
| MC.7a | Mother’s criminal behavior in childhood [4752] | 7 | 1 | 0 [0] | 1 | 0 [0] |
| MC.7b | Mother’s criminal behavior as teenager [4752] | 9 | 0 | 0 [0] | 2 | 2 [1] |
| **TOTAL** |  | **183** | **22** | **10 [0]** | **13** | **9 [1]** |

Note: the range of the number of valid observations by topic is shown in square brackets
