# Supplementary material for: Personality, Behavior and Environmental Features Associated with OXTR Genetic Variants in British Mothers
Source: PLoS One. 2014 Mar 12;9(3):e90465. doi: 10.1371/journal.pone.0090465 (PMC3951216; doi:10.1371/journal.pone.0090465)
Supplement: Table S4 — (DOCX) [file pone.0090465.s005.docx]

Table S4. The mother’s environment

|  |  |  | **rs53576** | | **rs2254298** | |
| --- | --- | --- | --- | --- | --- | --- |
| **Table Number** | **Topic** | **Number of Variables** | **<0.10** | **<0.05 [<0.01]** | **<0.10** | **<0.05 [<0.01]** |
| ME.1 | Maternal residence [7383-7550] | 7 | 0 | 0 [0] | 1 | 1 [0] |
| ME.2 | Housing circumstances [6107-7560] | 11 | 1 | 0 [0] | 0 | 0 [0] |
| ME.3 | Air pollutants  [7343-7560] | 14 | 0 | 0 [0] | 0 | 0 [0] |
| ME.4 | Pests and pets [7560] | 13 | 2 | 1 [0] | 0 | 0 [0] |
| ME.5 | Damp and mould [7440-7511] | 4 | 0 | 0 [0] | 1 | 1 [0] |
| ME.6 | Other features of home [7530-7560] | 9 | 0 | 0 [0] | 0 | 0 [0] |
| ME.7 | Domestic chemicals [7571] | 16 | 2 | 2 [1] | 2 | 2 [1] |
| ME.8 | Contact with chemicals as hobby or work [7611] | 17 | 3 | 3 [0] | 1 | 1 [1] |
| ME.9 | Specific types of work [6805-7564] | 9 | 2 | 1 [1] | 0 | 0 [0] |
| ME.10 | Electrical equipment [4658-7166] | 29 | 6 | 4 [1] | 4 | 3 [0] |
| ME.11 | Fluorescent lights [4828 – 7085] | 4 | 0 | 0 [0] | 0 | 0 [0] |
| ME.12 | Persons in household [6540-7611] | 18 | 1 | 0 [0] | 1 | 0 [0] |
| ME.13 | Social environment [6112-7677] | 25 | 1 | 1 [0] | 2 | 1 [0] |
| ME.14 | Physical activities and energy level pre-pregnancy [7080-7418] | 6 | 1 | 1 [0] | 3 | 2 [0] |
| ME.15 | Activity & energy in 1^st^ trimester [7065-7422] | 6 | 1 | 1 [0] | 2 | 1 [1] |
| ME.16 | Activity & energy in mid-pregnancy [6916-7418] | 25 | 0 | 0 [0] | 5 | 3 [2] |
| ME.17 | Activity & energy in late pregnancy [6643-7094] | 9 | 0 | 0 [0] | 0 | 0 [0] |
| ME.18 | Trace metals [2635-2726] | 8 | 2 | 0 [0] | 0 | 0 [0] |
| ME.19 | Dental care [3478-5905] | 9 | 0 | 0 [0] | 1 | 0 [0] |
| **TOTAL** |  | **239** | **22** | **14 [3]** | **23** | **15 [5]** |

Note: the range of the number of valid observations by topic is shown in square brackets
