# Supplementary material for: Personality, Behavior and Environmental Features Associated with OXTR Genetic Variants in British Mothers
Source: PLoS One. 2014 Mar 12;9(3):e90465. doi: 10.1371/journal.pone.0090465 (PMC3951216; doi:10.1371/journal.pone.0090465)
Supplement: Table S5 — (DOCX) [file pone.0090465.s006.docx]

Table S5. The mother prior to pregnancy

|  |  |  | **rs53576** | | **rs2254298** | |
| --- | --- | --- | --- | --- | --- | --- |
| **Table Number** | **Topic** | **Number of Variables** | **<0.10** | **<0.05 [<0.01]** | **<0.10** | **<0.05 [<0.01]** |
| MPB.1 | Biological/physiological measures [7724-7756] | 7 | 1 | 0 [0] | 1 | 1 [0] |
| MMH.1 | Medical history [7146-7282] | 13 | 2 | 0 [0] | 1 | 0 [0] |
| MMH.2 | History of surgery [7182-7266] | 6 | 0 | 0 [0] | 0 | 0 [0] |
| MMH.3 | History of injuries [7152-7295] | 17 | 1 | 0 [0] | 1 | 1 [0] |
| MMH.4 | History of allergies / atopic diseases [7124-7222] | 18 | 1 | 1 [0] | 2 | 1 [0] |
| MMH.5 | History of non-brain related diseases [7142-7295] | 10 | 2 | 1 [0] | 1 | 1 [0] |
| MMH.6 | History of neurological / psychiatric conditions [7251-7295] | 8 | 2 | 2 [0] | 0 | 0 [0] |
| MMH.7 | History of hearing and vision [7108-7295] | 11 | 0 | 0 [0] | 5 | 3 [0] |
| MMH.8 | History of hospital admissions [7295] | 1 | 0 | 0 [0] | 0 | 0 [0] |
| MMH.9 | History of X-rays [7295] | 19 | 3 | 2 [2] | 1 | 0 [0] |
| MPOH.1 | Reproductive background [6538-7277] | 8 | 0 | 0 [0] | 3 | 2 [1] |
| MPOH | Past obstetric history of mother [4210-7574] | 16 | 0 | 0 [0] | 1 | 1 [0] |
| **TOTAL** |  | **134** | **12** | **6 [2]** | **16** | **10 [1]** |

Note: the range of the number of valid observations by topic is shown in square brackets
