# Supplementary material for: Personality, Behavior and Environmental Features Associated with OXTR Genetic Variants in British Mothers
Source: PLoS One. 2014 Mar 12;9(3):e90465. doi: 10.1371/journal.pone.0090465 (PMC3951216; doi:10.1371/journal.pone.0090465)
Supplement: Table S6 — (DOCX) [file pone.0090465.s007.docx]

Table S6. The mother’s history of pregnancy

|  |  |  | **rs53576** | | **rs2254298** | |
| --- | --- | --- | --- | --- | --- | --- |
| **Table Number** | **Topic** | **Number of Variables** | **<0.10** | **<0.05 [<0.01]** | **<0.10** | **<0.05 [<0.01]** |
| MPB.2 | Biological measures in pregnancy [4819-6946] | 6 | 0 | 0 [0] | 0 | 0 [0] |
| MP1.1 | Signs and symptoms 1^st^ trimester [5911-7115] | 13 | 1 | 0 [0] | 1 | 0 [0] |
| MP1.2 | Procedures and tests 1^st^ trimester [7103-7114] | 6 | 0 | 0 [0] | 0 | 0 [0] |
| MP1.3 | Medication in 1^st^ trimester [7269-7530] | 17 | 2 | 1 [0] | 2 | 2 [0] |
| MP2.1 | Signs and symptoms mid-trimester [7060-7114] | 10 | 0 | 0 [0] | 1 | 1 [0] |
| MP2.2 | Procedures and tests mid-pregnancy [7103-7114] | 6 | 1 | 1 [0] | 0 | 0 [0] |
| MP2.3 | Medication in mid-pregnancy [7514-7530] | 16 | 2 | 1 [1] | 3 | 2 [0] |
| MP1H.1 | Hospital admissions [7072] | 1 | 1 | 0 [0] | 0 | 0 [0] |
| MP1.H2 | Medication 1^st^ half of pregnancy [7494-7574] | 12 | 4 | 2 [1] | 1 | 1 [0] |
| MP3.1 | Signs and symptoms 3^rd^ trimester [7113-7123] | 15 | 1 | 0 [0] | 1 | 0 [0] |
| MP3.2 | Procedures and tests 3^rd^ trimester [7123] | 6 | 0 | 0 [0] | 0 | 0 [0] |
| MP3.3 | Medication in 3^rd^ trimester [7073-7119] | 21 | 1 | 0 [0] | 2 | 1 [0] |
| MP3.4 | Supplements in 3^rd^ trimester [7098] | 6 | 0 | 0 [0] | 0 | 0 [0] |
| MP4.1 | Signs and symptoms last months [6870-6989] | 11 | 1 | 0 [0] | 0 | 0 [0] |
| MP4.2 | Procedures and tests last months [6989] | 3 | 0 | 0 [0] | 1 | 0 [0] |
| MP5.2 | X-rays in pregnancy [6079-7295] | 13 | 2 | 1 [0] | 4 | 3 [0] |
| **TOTAL** |  | **162** | **16** | **6 [2]** | **16** | **10 [0]** |

Note: the range of the number of valid observations by topic is shown in square brackets
