# Supplementary material for: Personality, Behavior and Environmental Features Associated with OXTR Genetic Variants in British Mothers
Source: PLoS One. 2014 Mar 12;9(3):e90465. doi: 10.1371/journal.pone.0090465 (PMC3951216; doi:10.1371/journal.pone.0090465)
Supplement: Table S7 — (DOCX) [file pone.0090465.s008.docx]

Table S7. Maternal diet and feeding habits

|  |  |  | **rs53576** | | **rs2254298** | |
| --- | --- | --- | --- | --- | --- | --- |
| **Table Number** | **Topic** | **Number of Variables** | **<0.10** | **<0.05 [<0.01]** | **<0.10** | **<0.05 [<0.01]** |
| MP5.1 | Eating disorders [7043-7104] | 6 | 3 | 2 [0] | 0 | 0 [0] |
| MP.DT1 | Dairy produce consumed [7114-7512] | 16 | 3 | 2 [0] | 4 | 2 [0] |
| MP.DT2 | Animal protein in diet [7114] | 10 | 2 | 1 [0] | 1 | 1 [0] |
| MP.DT3 | Vegetables and fruit [7114-7119] | 17 | 3 | 1 [0] | 0 | 0 [0] |
| MP.DT4 | Cereal based foods [7103-7119] | 12 | 2 | 2 [1] | 0 | 0 [0] |
| MP.DT5 | Fats used [6701-7100] | 15 | 1 | 1 [0] | 0 | 0 [0] |
| MP.DT6 | Other features of diet [7100-7119] | 5 | 1 | 1 [1] | 1 | 1 [0] |
| MP.DT7 | Snacks and sugar [7103-7128] | 7 | 0 | 0 [0] | 1 | 1 [0] |
| MP.DT8a | Basic nutrition [7096] | 8 | 4 | 4 [0] | 0 | 0 [0] |
| MP.DT8b | Specific amino and fatty acids [7126] | 8 | 3 | 3 [1] | 0 | 0 [0] |
| MP.DT8c | Vitamin content of diet [7096] | 12 | 2 | 2 [0] | 0 | 0 [0] |
| MP.DT8d | Trace elements in diet [7096] | 9 | 3 | 1 [1] | 0 | 0 [0] |
| MP.DT9 | Types of diet [7004-7209] | 5 | 2 | 2 [0] | 1 | 0 [0] |
| MP.DT10 | Dietary patterns [7111] | 5 | 0 | 0 [0] | 0 | 0 [0] |
| MP.DT11 | Herbal, organic and health foods [6607-7129] | 8 | 0 | 0 [0] | 1 | 1 [0] |
| **TOTAL** |  | **143** | **29** | **22 [4]** | **9** | **6 [0]** |

Note: the range of the number of valid observations by topic is shown in square brackets
