# Supplementary material for: Personality, Behavior and Environmental Features Associated with OXTR Genetic Variants in British Mothers
Source: PLoS One. 2014 Mar 12;9(3):e90465. doi: 10.1371/journal.pone.0090465 (PMC3951216; doi:10.1371/journal.pone.0090465)
Supplement: Table S8 — (DOCX) [file pone.0090465.s009.docx]

Table S8. Maternal exposures to noise and social drugs

|  |  |  | **rs53576** | | **rs2254298** | |
| --- | --- | --- | --- | --- | --- | --- |
| **Table Number** | **Topic** | **Number of Variables** | **<0.10** | **<0.05 [<0.01]** | **<0.10** | **<0.05 [<0.01]** |
| MN.1 | Noise [7035-7166] | 5 | 1 | 1 [0] | 2 | 2 [1] |
| MSD.1 | Active and passive cigarette smoking [5979-7574] | 29 | 13 | 4 [0] | 3 | 1 [1] |
| MSD.2 | Alcohol consumption [4482-7538] | 20 | 2 | 2 [0] | 3 | 2 [1] |
| MSD.3 | Caffeine containing drinks [4805-7528] | 16 | 1 | 1 [0] | 1 | 1 [0] |
| MSD.4 | Illicit drugs [7228-7476] | 4 | 0 | 0 [0] | 0 | 0 [0] |
| **TOTAL** |  | **74** | **17** | **8 [0]** | **9** | **6 [3]** |

Note: the range of the number of valid observations by topic is shown in square brackets
