# Supplementary material for: Personality, Behavior and Environmental Features Associated with OXTR Genetic Variants in British Mothers
Source: PLoS One. 2014 Mar 12;9(3):e90465. doi: 10.1371/journal.pone.0090465 (PMC3951216; doi:10.1371/journal.pone.0090465)
Supplement: Table S9 — (DOCX) [file pone.0090465.s010.docx]

Table S9. Maternal social exposure, moods and personality

|  |  |  | **rs53576** | | **rs2254298** | |
| --- | --- | --- | --- | --- | --- | --- |
| **Table Number** | **Topic** | **Number of Variables** | **<0.10** | **<0.05 [<0.01]** | **<0.10** | **<0.05 [<0.01]** |
| MSS.1 | Social status [5685-7559] | 3 | 1 | 0 [0] | 0 | 0 [0] |
| MSS.2 | Features of the neighbourhood [7358-7560] | 20 | 3 | 2 [0] | 2 | 2 [0] |
| MSS.3 | Criminal behaviour in adulthood [4752] | 9 | 2 | 0 [0] | 0 | 0 [0] |
| **TOTAL** | **Excluding moods and personality** | **32** | **6** | **2 [0]** | **2** | **2 [0]** |
|  |  |  |  |  |  |  |
| MPP.1 | Maternal moods during pregnancy [7500] | 12 | 0 | 0 [0] | 1 | 0 [0] |
| MPP.2 | Personality [6030-7723] | 11 | 1 | 1 [1] | 1 | 0 [0] |
| MPP.3 | Relationship with partner [7089] | 4 | 0 | 0 [0] | 0 | 0 [0] |
| MPP.4 | Religious belief [6410-7241] | 11 | 2 | 0 [0] | 1 | 1 [0] |
| MPP.5 | Attitudes and behaviour [7094-7140] | 8 | 0 | 0 [0] | 0 | 0 [0] |
| **TOTAL** | **Moods and personality** | **46** | **3** | **1 [1]** | **3** | **1 [0]** |

Note: the range of the number of valid observations by topic is shown in square brackets
