# Supplementary material for: Personality, Behavior and Environmental Features Associated with OXTR Genetic Variants in British Mothers
Source: PLoS One. 2014 Mar 12;9(3):e90465. doi: 10.1371/journal.pone.0090465 (PMC3951216; doi:10.1371/journal.pone.0090465)
Supplement: Table S10 — (DOCX) [file pone.0090465.s011.docx]

Table S10. Antenatal, labour and delivery features from obstetric records and maternal report

|  |  |  | **rs53576** | | **rs2254298** | |
| --- | --- | --- | --- | --- | --- | --- |
| **Table Number** | **Topic** | **Number of Variables** | **<0.10** | **<0.05 [<0.01]** | **<0.10** | **<0.05 [<0.01]** |
| MAN.1 | Antenatal blood pressures [4712-7817] | 4 | 0 | 0 [0] | 0 | 0 [0] |
| MAN.2 | Procedures recorded [4819] | 4 | 1 | 0 [0] | 0 | 0 [0] |
| MAN.3 | Organisation of care [4740-7817] | 6 | 0 | 0 [0] | 0 | 0 [0] |
| MAN.3a | Organisation – maternal report [6756-6992] | 8 | 0 | 0 [0] | 0 | 0 [0] |
| MAN.4 | Fetal lie and amniotic fluid [4766-4819] | 8 | 1 | 1 [0] | 2 | 0 [0] |
| MAN.5 | Maternal weight and weight gain [7785] | 4 | 1 | 0 [0] | 1 | 0 [0] |
| MAN.7 | Bleeding and haemoglobin [4819-6573] | 8 | 0 | 0 [0] | 1 | 1 [0] |
| MAN.8 | Infections and other morbidity [4819-7817] | 6 | 0 | 0 [0] | 0 | 0 [0] |
| MLD.1 | Details of labour and delivery [3888-4819] | 14 | 1 | 0 [0] | 3 | 2 [2] |
| MLD.1a | Details – maternal report [6491-7012] | 16 | 0 | 0 [0] | 0 | 0 [0] |
| MLD.2 | Induction and augmentation [4223-4819] | 8 | 2 | 1 [0] | 1 | 1 [0] |
| MLD.3 | Analgesics and anaesthetics [4727-4819] | 14 | 1 | 0 [0] | 3 | 2 [1] |
| MLD.3a | Maternal report of analgesics [7012] | 5 | 0 | 0 [0] | 0 | 0 [0] |
| MLD.4 | Medication in labour [4704-4819] | 10 | 0 | 0 [0] | 2 | 0 [0] |
| MLD.5 | Signs and symptoms in labour [3225-4819] | 9 | 2 | 0 [0] | 0 | 0 [0] |
| MLD.6 | Reaction of mother to labour/delivery [6222-6911] | 8 | 3 | 3 [1] | 2 | 1 [0] |
| **TOTAL** |  | **132** | **12** | **5 [1]** | **15** | **7 [3]** |

Note: the range of the number of valid observations by topic is shown in square brackets
