# Supplementary material for: Personality, Behavior and Environmental Features Associated with OXTR Genetic Variants in British Mothers
Source: PLoS One. 2014 Mar 12;9(3):e90465. doi: 10.1371/journal.pone.0090465 (PMC3951216; doi:10.1371/journal.pone.0090465)
Supplement: Table S11 — (DOCX) [file pone.0090465.s012.docx]

Table S11. Maternal social exposure, moods and personality

|  |  |  | **rs53576** | | **rs2254298** | |
| --- | --- | --- | --- | --- | --- | --- |
| **Table Number** | **Topic** | **Number of Variables** | **<0.10** | **<0.05 [<0.01]** | **<0.10** | **<0.05 [<0.01]** |
| CN.1 | The fetus/baby in labour and at birth [4152-4819] | 50 | 5 | 2 [0] | 3 | 1 [0] |
| CN.2 | Investigations and treatment [4339-4702] | 14 | 6 | 2 [1] | 3 | 0 [0] |
| CN.3 | Neonatal signs and symptoms [4413-4820] | 11 | 1 | 0 [0] | 2 | 1 [0] |
| CN.4 | Medication to neonate [4412-4702] | 4 | 0 | 0 [0] | 1 | 1 [0] |
| CN.5 | Outcome of pregnancy [7814-7891] | 11 | 0 | 0 [0] | 3 | 2 [0] |
| **TOTAL** |  | **90** | **12** | **4 [1]** | **12** | **5 [0]** |

Note: the range of the number of valid observations by topic is shown in square brackets
