# Supplementary material for: Personality, Behavior and Environmental Features Associated with OXTR Genetic Variants in British Mothers
Source: PLoS One. 2014 Mar 12;9(3):e90465. doi: 10.1371/journal.pone.0090465 (PMC3951216; doi:10.1371/journal.pone.0090465)
Supplement: Table S12 — (DOCX) [file pone.0090465.s013.docx]

Table S12. Comparison of daily nutrient intake of GG women compared with those with an A allele on rs53576 – those with P <0.10 listed, after omitting all with a history of bulimic type behaviour (n = 6536)

| **Nutrient** | **Effect size [95%CI]** | **P value** |
| --- | --- | --- |
| Non-milk extrinsic sugar (g) | -2.39 [-4.02, -0.76] | 0.004 |
| Sugar (g) | -2.73 [-4.57, -0.90] | 0.004 |
| Carbohydrate (g) | -2.76 [-5.72, +0.20] | 0.068 |
| Energy (kJ) | -103.6 [-199.1, -8.11] | 0.033 |
| Fat (g) | -1.36 [-2.47, -0.24] | 0.017 |
| Monounsaturated fat (g) | -0.46 [-0.84, -0.08] | 0.019 |
| Saturated fat (g) | -0.71 [-1.27, -0.16] | 0.012 |
| Cholesterol (mg) | -6.06 [-10.3, -1.85] | 0.005 |
| Vitamin B12 (µg) | -0.16 [-0.29, -0.03] | 0.013 |
| Retinol (µg) | -22.6 [-39.0, -6.29] | 0.007 |
| Riboflavin (mg) | -0.03 [-0.05, -0.00] | 0.048 |
| Calcium (mg) | -15.2 [-28.9, -1.62] | 0.028 |
| Potassium (mg) | -29.7 [-64.5, +5.12] | 0.095 |
| Phosphorus (mg) | -14.8 [-31.4, +1.79] | 0.080 |
| Iodine (µg) | -3.34 [-5.64, -1.05] | 0.004 |
